# Bacteroides fragilis RecA protein overexpression causes resistance to metronidazole

**DOI:** 10.1016/j.resmic.2010.04.003

**Published:** 2010-06

**Authors:** Laura S. Steffens, Samantha Nicholson, Lynthia V. Paul, Carl Erik Nord, Sheila Patrick, Valerie R. Abratt

**Affiliations:** a Department of Molecular and Cell Biology, University of Cape Town, Rondebosch, Private Bag, Cape Town 7701, South Africa; b Karolinska Institute, Department of Laboratory Medicine, Division of Clinical Microbiology, Karolinska University Hospital, Stockholm, Sweden; c Centre for Infection and Immunity, School of Medicine, Dentistry and Biomedical Sciences, Queen’s University, Belfast, UK

**Keywords:** *Bacteroides fragilis*, RecA, Metronidazole, Hydrogen peroxide, Ultraviolet radiation

## Abstract

*Bacteroides fragilis* is a human gut commensal and an opportunistic pathogen causing anaerobic abscesses and bacteraemias which are treated with metronidazole (Mtz), a DNA damaging agent. This study examined the role of the DNA repair protein, RecA, in maintaining endogenous DNA stability and its contribution to resistance to Mtz and other DNA damaging agents. RT-PCR of *B. fragilis* genomic DNA showed that the *recA* gene was co-transcribed as an operon together with two upstream genes, putatively involved in repairing oxygen damage. A *B. fragilis recA* mutant was generated using targeted gene inactivation. Fluorescence microscopy using DAPI staining revealed increased numbers of mutant cells with reduced intact double-stranded DNA. Alkaline gel electrophoresis of the *recA* mutant DNA showed increased amounts of strand breaks under normal growth conditions, and the *recA* mutant also showed less spontaneous mutagenesis relative to the wild type strain. The *recA* mutant was sensitive to Mtz, ultraviolet light and hydrogen peroxide. A *B. fragilis* strain overexpressing the RecA protein exhibited increased resistance to Mtz compared to the wild type. This is the first study to show that overexpression of a DNA repair protein in *B. fragilis* increases Mtz resistance. This represents a novel drug resistance mechanism in this bacterium.

## 1 Introduction

*Bacteroides* species are amongst the earliest commensals to colonise the gut, accounting for approximately 30% of colonic symbionts, and *Bacteroides fragilis* is an opportunistic pathogen (Patrick, 2002; Patrick and Duerden, 2006) causing approximately half of anaerobic bacteraemia, 19% of which are potentially fatal (Sears, 2001). Metronidazole (Mtz) is the preferred antibiotic for treating anaerobic infections (Haggoud et al., 1994), and it exerts a bactericidal effect by generating single-stranded (ss) and double-stranded (ds) DNA breaks (Trinh and Reysset, 1998; Sisson et al., 2000). Nonetheless, the emergence of Mtz resistance mechanisms has increasingly compromised the effectiveness of treatment (Chang et al., 1997; Wareham et al., 2005). A wide range of Mtz resistance mechanisms have been described in *B. fragilis*. These include decreased activity or total inactivation of electron transport chain components (Diniz et al., 2004), overexpression of multidrug efflux pumps (Pumbwe et al., 2007) and the expression of 5-nitroimidazole nitroreductases (encoded by *nim* genes) that convert Mtz to non-toxic amino derivatives (Diniz et al., 2004). In addition, overexpression of the rhamnose regulatory protein RhaR is linked with Mtz resistance in *Bacteroides thetaiotaomicron* (Patel et al., 2009) and the *reg* gene (BF3248) of *B. fragilis*, a member of the AraC family, is also involved in resistance to Mtz and other DNA damaging agents (Casanueva et al., 2008). A number of Mtz-resistant clinical isolates, however, do not contain *nim* genes or any of the previously described resistance mechanisms. Since Mtz exerts its bactericidal effect through generating DNA strand breaks, the possible role of DNA repair proteins in the response to treatment with Mtz is of interest (Chang et al., 1997).

RecA is a major DNA repair protein which carries out homologous recombination repair and controls the expression of many other DNA repair proteins in certain bacterial species through the SOS response (Kuzminov, 1999). The *B. fragilis recA* gene has previously been cloned and functionally characterised (Goodman et al., 1987; Goodman and Woods, 1990). The *recA* genomic context showed that it clustered with genes possibly implicated in cellular responses to oxidative stress. This suggested a novel mechanism for the coupling of antioxidant- and RecA-mediated DNA repair processes. The aims of this study were to analyse the genetic context of the *B. fragilis recA* gene, to generate a *B. fragilis recA* mutant and to examine its phenotype with regard to DNA damaging agents including Mtz. In addition, overexpression of the RecA protein in *B. fragilis* was examined to ascertain whether enhanced DNA repair could cause Mtz resistance.

## 2 Methods

### 2.1 Bioinformatic analysis

The bacterial strains used for bioinformatic analysis are shown in Table 1. Protein and DNA sequences were obtained from the National Centre for Biotechnology Information (www.ncbi.nih.gov), except for the sequences for *B. fragilis* 638R which were produced by the *B. fragilis* Sequencing Group at the Sanger Institute (ftp://ftp.sanger.ac.uk/pub/pathogens/bf/BF638R.dbs). BLAST 2.2.17 was used to calculate the predicted percentage identity between protein sequences (Altschul et al., 1997). Conserved domains database (CDD) searches were used to identify conserved domains in the protein sequences (Table 2) (Marchler-Bauer and Bryant, 2004). Protein sequence alignments were carried out with DNAMAN version 4.13 (Lynnon BioSoft).

### 2.2 Bacterial strains and plasmids, media and growth conditions

The strains and plasmids used are described in Table 1. *Escherichia coli* strains were grown in Luria–Bertani (LB) broth or on LB plates under aerobic conditions at 37 °C (Maniatis et al., 1982). *E. coli* cells harbouring plasmids were grown in LB supplemented with 100 mg/L ampicillin (amp). *B. fragilis* 638R strains were grown in supplemented brain heart infusion broth (BHISB) or on plates (BHISA) at 37 °C under anaerobic conditions (Holdeman and Moore, 1972). *B. fragilis* mutants were grown on BHISA including erythromycin (erm; 10 mg/L), while *B. fragilis* cells containing pLYL01 or pLYrecA were grown on BHISA supplemented with tetracycline (tet; 2 mg/L).

### 2.3 Transcriptional analysis of a putative operon

RNA was isolated from *B. fragilis* cells grown to log phase (OD_600_ 0.6) in BHISB using the method of Aiba et al. (1981), with additional purification using the Qiagen RNAEasy Mini Kit (Qiagen). The cDNA synthesis was carried out using the First Strand cDNA Synthesis kit (Fermentas). Amplification of the intergenic regions was done using primers pairs FBRT–RBRT for genes BF638R1248 and BF638R1246/7, and pairs FRA–RART for BF638R1246/7 and BF638R1245 (Table 3). The PCR parameters were: initial denaturation of 95 °C for 5 min, then 25 cycles of denaturation at 95 °C for 30 s, annealing at 53.8 °C for 30 s and elongation at 72 °C for 3 min. A final elongation step was carried out at 72 °C for 5 min.

### 2.4 DNA techniques and construction of *B. fragilis* derivative strains

*B. fragilis* genomic DNA was extracted according to Dachs et al. (1995). All cloning reagents and restriction enzymes were purchased from Fermentas. Plasmids were transformed into electrocompetent *E. coli* cells using electroporation parameters of 2.5 kv, 200 Ω and 25 μF (Tung and Chow, 1995). For generating a *B. fragilis recA* insertional mutant, a *B. fragilis recA* internal fragment was obtained by PCR using primer pair RIF–RIR specific for BF638R1245 (Table 3). The PCR parameters were as described previously, except that the annealing temperature was 53 °C. The *recA* internal fragment was cloned into the pGERM *Sma*I site to generate pGREC, which was then transformed into *E. coli* S17-1. Mating of *E. coli* S17-1 and *B. fragilis* was performed (Shoemaker et al., 2000) and single colonies were analysed to confirm the mutation using PCR and primer pairs FRA-M13R and RRA-M13F as described previously. The PCR product was sequenced to confirm its identity. For complementing the *recA* mutant and overexpressing *recA* in *B. fragilis*, electrocompetent *E. coli* S17-1 cells were transformed with pLYL01 or pLYLrecA, which had a full-length copy of the *recA* gene cloned into the *Pst*I and *Sph*I sites with the primers FRA and RRA (Table 3), and transferred to *B. fragilis* as previously described. Transconjugants were plated onto BHISA containing tetracycline (2 mg/L) and gentamicin (200 mg/L). Single colonies were analysed using PCR to confirm the presence of the plasmids.

### 2.5 Cell morphology and DNA strand break analysis

*B. fragilis* 638R and *B. fragilis recA* were subcultured on BHISA and washed in phosphate-buffered saline (PBS) buffer pH 7.4. For nuclear staining, 4,6-diamidino-2-phenylindole dihydrochloride (DAPI) (Sigma–Aldrich) at 1 mg/L was used. For membrane staining, FM-4-64 (Sigma–Aldrich) was applied at 1 mM/ml. The stains were added directly to the cell suspensions, incubated on ice for 15 min, and washed and resuspended in PBS. The resuspended cells were placed on acid-washed slides (Spector and Goldman, 2006), dried at 65 °C and covered with Mowiol pretreated with n-propylgallate (Sigma–Aldrich). A glass coverslip was placed over the sample and the slides were visualised using fluorescence microscopy at 1000× magnification on the Zeiss Axiovert 200 and photographed using Zeiss Axiocam HR and Axiovision 4.6 software. Images were separated into quadrants, the cell numbers exhibiting atypical DAPI staining were counted, and the percentage occurrence of these, with reference to the total number of cells, was calculated. Gram staining and conventional light microscopy (Leitz Diaplan light microscope at a magnification of 1000×) were also used. Microscope photographs were captured by a Zeiss Axiocam camera and visualised with Axiovision 2.0.5.3. This experiment was carried out in technical duplicate and biological triplicate, and a standard student two-tailed *T*-test was used to determine the statistical significance.

Denaturing gel electrophoresis was performed to investigate DNA strand breaks (Abratt et al., 1986) and the gel was visualised with short wavelength UV light using GelDoc (BioRad) and photographed.

### 2.6 Spontaneous mutation analysis

The effect of a mutation in the *recA* gene on the basal mutation rate was measured using the generation of spontaneous resistance to fusidic acid (Fung-Tomc et al., 1993). *B. fragilis* 638R (pLYL01)*, B. fragilis* 638R *recA* (pLYL01) and *B. fragilis* 638R *recA* (pLYLRecA) were grown for 16 h in BHISB. Cells (100 μl aliquots) were plated on each of 10 BHISA plates, without l-cysteine, in the presence or absence of 6 mg/L fusidic acid (Sigma–Aldrich) and incubated at 37 °C for 3 days under anaerobic conditions. The mutation rate was calculated by determining the number of surviving cells/colony forming units per ml of original cells plated. All experiments were completed as biological and technical triplicates and a Student’s *T*-test was used to establish the statistical significance of the results.

### 2.7 Growth of *B. fragilis* strains and cell survival in the presence of DNA damaging agents

Cultures of *B. fragilis* 638R (pLYL01), 638R*recA* (pLYL01), 638R (pLYLRecA) and 638R*recA* (pLYLRecA) were incubated anaerobically for 16 h at 37 °C in BHISB and then exposed to three different DNA damaging agents under strict anaerobic conditions.

For exposure to UV light (254 nm), the 16 h culture was diluted 100-fold in water, exposed to varying doses of UV radiation, and diluted and plated on BHISA. For Mtz exposure, the 16 h culture was grown to log phase in BHISB (OD_600_ = 0.6) and exposed to 5 mg/L Mtz. Cell samples were collected at 15 min intervals, diluted as before and plated on BHISA. For hydrogen peroxide exposure, the 16 h culture was similarly grown to log phase in BHISB (OD_600_ = 0.6). One millilitre of the culture was removed, centrifuged and the pellet resuspended in PBS pH 7.4 and hydrogen peroxide (Sigma–Aldrich) was added to a final concentration of 73 μM. Cells were sampled at 5 min intervals for 15 min and plated on BHISA (without l-cysteine). For all treatments, the plates were incubated anaerobically at 37 °C for 3 days and the surviving fraction of cells was calculated for each time point. All experiments were done in triplicate (Sund et al., 2008). In addition, the Mtz susceptibility of the strains was determined by measuring the minimum inhibitory concentration (MIC) on BHISA plates using E-strips according to the manufacturers’ instructions (AB Biodisk).

## 3 Results

### 3.1 Bioinformatic analysis of *B. fragilis recA* and its upstream genes

Bioinformatic analysis was carried out by performing a multiple sequence alignment of the deduced amino acid sequences of the RecA proteins from *B. fragilis* 638R and the other bacteria listed in Table 1. The *B. fragilis* 638R RecA protein exhibited a predicted amino acid identity of 93% to *B. thetaiotaomicron*, 81% to *Porphyromonas gingivalis*, 69% to *Bacillus subtilis*, 62% to *E. coli* and 61% to *Deinococcus radiodurans* RecA proteins as calculated by BLAST analysis (Altschul et al., 1997). Like the *E. coli* RecA, *B. fragilis* RecA showed high conservation of Walker A (GPESSGKT) and Walker B (IIVD) (Table 2) which are signature motifs of ATP binding domains (Marchler-Bauer and Bryant, 2004). Important residues for ATP binding are the glutamate (E) and glutamine (Q) (Bell, 2005). The L1 and L2 motifs are involved in the binding of ssDNA (Chen et al., 2007) and the sequence alignment showed a high degree of conservation of L1 and L2 between the analysed bacteria.

**Fig. 1:**
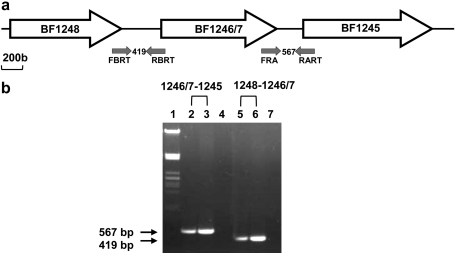
(a) Genetic context of *B. fragilis* 638R *recA* and RT-PCR primer combinations. The primers shown above are fully described in Table 3. Grey arrows, primers amplifying intergenic regions. (b) RT-PCR of intergenic regions indicated, using RNA extracted from *B. fragilis* 638R. Lane 1, Molecular size marker (λ DNA digested with *Pst*I); lanes 4 & 7, no DNA template control; lanes 2 & 5, cDNA; lanes 3 & 6, genomic DNA.

Scrutiny of the arrangement of the genes adjacent to the *B. fragilis* 638R *recA* (BF638R1245) revealed that it could be part of an operon along with BF638R1246/7 and BF638R1248 (Fig. 1A). There are 82 bp between BF638R1248 and BF638R1246/7 and 97 bp between BF638R1246/7 and BF638R1245. An investigation was carried out using a conserved domain database (CDD) search to determine whether the genes flanking *B. fragilis* 638R *recA*-encoded proteins possibly related to RecA function. The hypothetical protein product of BF638R1248 was found to contain a homospermidine synthase domain (Marchler-Bauer and Bryant, 2004), which catalyses the synthesis of polyamine homospermidine from putrescine (Tholl et al., 1996). BF638R1246 has been annotated as encoding a putative thiol-specific antioxidant (TSA) enzyme, while BF638R1247 has been annotated as encoding a bacterioferritin comigratory protein (BCP). The annotation in *B. fragilis* 638R is different from that for *B. fragilis* NCTC 9343 and YCH46, where BCP and TSA are classified as one gene with the BF638R1246 start site.

A cDNA conversion was carried out on DNA-free RNA extracted from exponential phase *B. fragilis* 638R cultures under normal growth conditions. Primer pairs to the intergenic regions produced PCR products from the cDNA template (Fig. 1B), indicating that the three-gene cluster is transcribed as an operon. Wild type genomic DNA was used as a positive control, while no product was obtained when RNA was used as the template as a negative control (results not shown).

### 3.2 Insertional inactivation of *B. fragilis recA* and genetic confirmation of the mutant

To test the function of RecA in *B. fragilis,* a *recA* mutant was generated using targeted gene disruption via the suicide vector pGREC (Table 1). PCR was performed on the transconjugants to verify the mutation. Primers FRA–RRA generated a 1.6 kb PCR product from wild type DNA, but not in the putative mutant indicating disruption of *recA* (results not shown). The mutant produced an 838 bp product with PCR primers FRA and M13R and an 818 bp fragment from RRA and M13F, respectively confirming the insertion of the pGREC plasmid within the *recA* gene.

### 3.3 *B. fragilis recA* mutant cell morphology

The cellular morphologies of *B. fragilis* wild type and *recA* mutant strains were investigated using fluorescence microscopy coupled with a nucleophilic dye (DAPI) to detect the nuclear material and a lipophilic dye (FM-4-64) to visualise the membrane. There was no significant elongation of the mutant strain under normal growth conditions. The mutant strain, however, showed a statistically significant increase in the proportion of cells where DAPI staining of the DNA was absent or reduced as compared to the wild type (43.95% vs 2.33% respectively; *p* = 0.00009). DAPI binds to double-stranded DNA fragments and fluoresces in a concentration-dependent manner (Breusegem et al., 2002). The results therefore indicate that the cells that have reduced DAPI staining either have a very high proportion of damaged and fragmented DNA, or have reduced amounts of nuclear material.

### 3.4 DNA strand break analysis

**Fig. 2:**
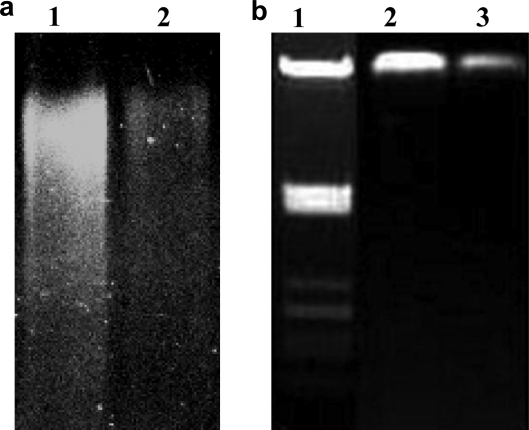
Determination of DNA breaks. (a) Alkaline denaturing agarose gel electrophoresis (0.5%) of *B. fragilis* 638R (lane 1) and the *recA* mutant (lane 2). (b) Agarose gel electrophoresis (0.8%) of lane 1, molecular size marker (λ DNA digested with *Pst*I); *B. fragilis* 638R (lane 2) and *recA* mutant (lane 3).

In order to further investigate the link between DNA damage and cellular division, the extent of DNA damage in both strains under normal growth conditions was evaluated by alkaline denaturing gel electrophoresis of equivalent concentrations of DNA extracted from the *B. fragilis* wild type and *recA* mutant (Fig. 2). The DNA from both strains was of high molecular mass when electrophoresed under non-denaturing conditions, although the amount of high molecular weight DNA in the mutant appeared to be slightly reduced, indicating a low level of double-strand breaks. The denaturing gels, however, showed a marked difference in the DNA between the wild type and mutant strains. The majority of genomic DNA in the wild type was high molecular mass with very little degradation. In contrast, the mutant strain exhibited considerable DNA degradation and reduced high molecular mass DNA, indicating the presence of single-strand breaks. This supports the hypothesis that there is an accumulation of DNA strand breaks in the *recA* mutant. When taken in conjunction with the fluorescence microscopy results, the denaturing gel result supports the hypothesis that RecA is involved in the maintenance of DNA integrity under normal growth conditions.

### 3.5 Mutation rate analysis

The spontaneous mutation rate of *B. fragilis* wild type and *recA* mutant was established by measuring the number of fusidic-acid-resistant survivors for each strain. The mutation rate in the wild type strain was 1.12 × 10^−9^ while the *recA* mutant strain had a mutation rate of only 5.85 × 10^−10^, a statistically significant twofold reduction (*p*-value of 0.000568) when compared to the wild type. These results support a potential role for RecA in mutagenesis in *B. fragilis*.

### 3.6 Cell survival in response to DNA damaging agents

**Fig. 3:**
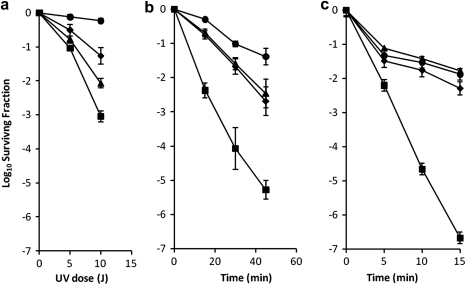
Survival curves of the *B. fragilis* strains in response to DNA damage with (a) UV, (b) Mtz and (c) hydrogen peroxide. Filled circles, *B. fragilis* 638R(pLYL01); filled squares, *B. fragilis* 638R *recA^-^* mutant(pLYL01); filled triangles, *B. fragilis* 638R *recA^-^* mutant complemented with pLYLrecA; filled diamonds, *B. fragilis* 638R *recA* overexpressor (pLYLrecA). The errors bars represent the standard error calculated from at least three replicates of data.

The effects of UV, Mtz and H_2_O_2_ on the viability of the *B. fragilis recA* mutant (638R *recA* (pLYL01), a complemented strain (638R *recA* (pLYLrecA) and the parental strain containing the *rec A* gene on a plasmid (638R (pLYLrecA) (RecA overexpressor) were examined. The *recA* mutant showed a 2 log_10_ decrease in survival in the presence of UV compared to wild type cells (Fig. 3A), indicating *B. fragilis* RecA involvement in repairing UV-induced thymine dimers. The *recA* mutant strain, complemented with the functional *recA* gene on a plasmid, did not fully regain the wild type phenotype but it did have increased survival compared to the mutant (Fig. 3A). In *B. fragilis* 638R, overexpression of the RecA protein, introduced on plasmid pLYLrecA into the wild type strain did, however, result in increased survival of the transconjugant as compared to the wild type strain carrying the same plasmid with no *recA* gene inserted.

*B. fragilis* wild type and *recA* mutant cells were exposed to Mtz (Fig. 3B). The *B. fragilis* 638R *recA* mutant exhibited a 2-log_10_ decrease in survival after 45 min compared to that of the wild type cells. *B. fragilis* RecA is therefore involved in repairing the DNA strand breaks caused by Mtz. This result is similar to that observed in the *recA* mutants of *B. thetaiotaomicron* (Cooper et al., 1997). The complemented *B. fragilis* 638R *recA* mutant regained the full wild type phenotype in the presence of Mtz (Fig. 3B), unlike the result seen for UV. As was seen for UV survival, overexpression of RecA in the wild type *B. fragilis* cells caused improved survival when they were challenged with Mtz. The MIC plate results confirmed these findings, except that the method was not sensitive enough to detect the increase in Mtz resistance in cells overexpressing RecA (Table 4).

The *B. fragilis* wild type and *recA* mutant strains were exposed to 73 μM hydrogen peroxide for 15 min (Fig. 3C). The *recA* mutant exhibited a 5 log_10_ decrease in survival compared to the wild type, and complementation with pLYLRecA led to full recovery of the wild type phenotype (Fig. 3C). Overexpression of the RecA protein did not cause an increase in the ability of the cells to recover from lethal doses of hydrogen peroxide.

## 4 Discussion

In *B. fragilis*, the close proximity of putative oxidative stress genes to *recA* could allow for an efficient coordinated response to oxidative stress as well as DNA damage, since both Mtz and oxidative stress conditions cause DNA strand breaks (Trinh and Reysset, 1998; Sund et al., 2008). Three other TSA peroxidases have previously been identified in *B. fragilis*: alkyl hydroperoxide peroxidase (AhpC), BCP and thioredoxin peroxidase (Tpx) (Chae et al., 1994; Herren et al., 2003; Chen et al., 2007). TSA peroxidases reduce peroxides to alcohols with the aid of a reduced thiol donor (Herren et al., 2003; Chen et al., 2007). AhpC/TSA enzymes have been identified in four opportunistic pathogens, namely *Enterococcus histolytica*, *Helicobacter pylori*, *Cryptosporidium parvum* and *B. fragilis* (Chae et al., 1994). These enzymes may provide protection against the oxidative burst generated by macrophages and neutrophils during the host immune response to infection. In *E. coli*, polyamines and polyamine synthesis enzymes have been found to affect gene expression under oxidative stress (Jung and Kim, 2003).

The *recA* gene has been shown to form part of an operon in numerous other bacterial species; however, none of these has been linked to the oxidative stress response as is found in *B. fragilis*. In both *Mycobacterium smegmatis* and *Streptomyces lividans*, *recA* and *recX* are co-transcribed as an operon (Vierling et al., 2000). In *S. lividans*, the operon is only transcribed in the presence of DNA damage, while *recA* is constitutively expressed at basal levels under non-inducing conditions. This differs from *M. smegmatis,* where both genes are expressed jointly at all times. RecX is thought to bind the nucleoprotein filament which leads to disassembly of RecA from the DNA during recombination; thus it functions as a negative regulator of RecA activity (Lusetti et al., 2004). Consequently, RecX protects the cell from RecA overexpression toxicity (Vierling et al., 2000). In *B. fragilis* NCTC 9343, the protein product of BF0454 is annotated as being a putative transcriptional regulator with limited similarity to *Pseudomonas aeruginosa* RecX; however, BF0454 is not clustered with *recA* on the genome. The *recA* gene in *D. radiodurans* forms an operon with *cinA* and *ligT* (Bonacossa de Almeida et al., 2002), while in *Streptococcus pneumoniae*, *recA* forms an operon with *cinA*, *dinF* and *lytA* (Mortier-Barriere et al., 1998). The *cinA* gene is a competence-induced gene and might encode a recombination accessory protein (Bonacossa de Almeida et al., 2002). The *ligT* gene encodes a 3′–5′ DNA ligase (Bonacossa de Almeida et al., 2002), *dinF* codes for a multidrug efflux pump in *Ralstonia solancearum* (Brown et al., 2007) and *lytA* codes for the pneumococcal autolysin (Mortier-Barriere et al., 1998). The *recA* operon in *B. fragilis* therefore presents a novel operon arrangement with the *B. fragilis recA* gene clustered with putative oxidative stress response genes.

In both Gram-positive and Gram-negative bacteria, there is an established link between the DNA integrity of a cell and cellular division (O’Reilly and Kreuzer, 2004). A change in the integrity of the nuclear material of the cell is usually indicated by cellular elongation due to a halt in cell division (Hill et al., 1997; O’Reilly and Kreuzer, 2004). This is well characterised in *E. coli* and *B. subtilis* as a RecA-mediated SOS response. The coupling of the cell cycle to DNA damage has also been reported as a RecA-independent process (Hill et al., 1997; Goranov et al., 2005).

In *B. fragilis* there is as yet no known SOS-like response and for this reason it was important to establish in this study whether there was a RecA-dependent link between the cell cycle and the replicative status of the DNA in the cell. The *B. fragilis* RecA mutant exhibited an unusual distribution of the DNA following cell division. The reason for this may be that the cells do not divide as frequently if there is a high proportion of damaged DNA (O’Reilly and Kreuzer, 2004). The decrease in the number of these cells in the wild type suggests that, in *B. fragilis,* functional RecA plays a role in maintaining the nuclear material and may restart cellular division in response to repaired DNA damage. This type of atypical cell division has been linked to RecA in *B. subtilis* amongst others (Sciochetti et al., 2001) where RecA mutant cells have been shown to inherit no nuclear material after division.

The induction of the SOS response and the repair of ssDNA breaks have been linked to the replicative status of the cell in *E. coli* and *B. subtilis.* The role of the replisome is undefined in this process. The redistribution of the RecA protein to ssDNA breaks seems to require the presence of the replisome in both of these model systems (Simmons et al., 2007). These findings led to the hypothesis that in the RecA-deficient system of the mutant, replication is undertaken to facilitate the redistribution of the absent RecA protein. This replication leads to cell division and explains the atypical DAPI staining as well as the increased occurrence of ssDNA in the *recA* mutant.

The response of individual cells to DNA damage can either be a highly accurate or a mutagenic repair process (Sweasy et al., 1990). In *E. coli,* the mutagenic repair process is an SOS-associated pathway (Kuzminov, 1999), the main components of which are the UmuC and UmuD proteins that form the subunits for polymerase V, an error-prone DNA polymerase with no proofreading function (Sweasy et al., 1990). The initiation of translesion synthesis by the UmuCD polymerase across damaged regions of DNA is controlled by the coprotease activity of the RecA protein (O’Reilly and Kreuzer, 2004) and allows for mutagenic repair of the DNA damage (Kuzminov, 1999). The *B. fragilis* genome shows the presence of putative proteins with sequence similarity to both the UmuC (BF1863 YCH46) and D (BF1928 YCH46) subunits of DNA polymerase V. This supports the hypothesis that *B. fragilis* may possess a mutagenic repair pathway facilitated by RecA which could be similar to the UmuCD pathway in *E. coli*. This pathway would be inactive in the absence of RecA and this could explain the reduction in the mutation rate observed in the *recA* mutant when exposed to fusidic acid stress.

The ability of cells to survive oxidative stress is due in large part to the ability of a cell to repair the DNA damage induced by the reactive oxygen species introduced into the system (Imlay, 2002). These results indicate a strong link between the cell’s ability to survive oxidative stress and the RecA-mediated repair pathway.

This is the first study to report that overexpression of a major DNA repair protein in *B. fragilis* can lead to increased resistance to UV radiation and Mtz treatment (Fig. 3A, B). It also shows a convincing link between the recombinatorial repair process in this bacterium and survival in the face of oxidative stress (Fig. 3C). The results suggest that the regulatory mechanisms for the RecA protein differ in response to the various types of damage investigated, as shown by the incomplete complementation in response to UV exposure and the full in trans complementation of RecA function following exposure to Mtz and hydrogen peroxide. Incomplete complementation has also seen in a complemented *Enterococcus faecalis recA* mutant (Weaver and Reddy, 2006). The authors attributed this to unknown effects on gene expression due to the complemented copy being on a plasmid and not integrated into the chromosome. The absence of increased survival of *B. fragilis* after hydrogen peroxide exposure even in the presence of excess RecA suggests that the upregulation of the adjacent genes may be required. This will be investigated in further work, along with the overexpression of other DNA repair proteins to see if a similar Mtz resistance phenotype is seen.

## Figures and Tables

**Table 1:** Bacterial strains and plasmids.

Strain/plasmid	Relevant characteristics or use	Source/reference
*Bacillus subtilis* subsp*. subtilis* str. 168	Multiple sequence alignment	NC_000964
*Bacteroides fragilis* NCTC 9343	Multiple Sequence alignment	NC_003228
*Bacteroides thetaiotoamicron* VP1-5482	Multiple sequence alignment	NC_004663
*Deinococcus radiodurans* R1	Multiple sequence alignment	NC_001263
*Escherichia coli* K-12	Multiple sequence alignment	NC_000913
*Porphyromonas gingivalis* W83	Multiple sequence alignment	NC_002950
*Bacteroides fragilis 638R*	Multiple sequence alignment	Privitera et al., 1979

*Bacteroides fragilis*		
638R	Clinical strain, Rif^R^Gent^R^	Privitera et al., 1979
638R *recA*	638R derivative, *recA* Rif^R^Gent^R^Erm^R^	This study
638R (pLYL01)	638R Rif^R^Gent^R^Tet^R^	This study
638R *recA* (pLYL01)	638R *recA* Rif^R^Gent^R^Erm^R^Tet^R^	This study
638R *recA*(pLYLrecA)	638R *recA* (*recA*^+^) Rif^R^Gent^R^Erm^R^Tet^R^	This study
638R (pLYLrecA)	638R (*recA*^+^) Rif^R^ Gent^R^ Tet^R^	This study

*Escherichia coli*		
S17-1	RP4-2-Tc::Mu *aph*::Tn*7 recA* Strep^R^	Simon et al., 1983
S17-1 (pGREC)	S17-1 containing pGREC	This study
S17-1 (pLYL01)	S17-1 containing pLYL01	This study
S17-1 (pLYLrecA)	S17-1 containing pLYLrecA	This study

Plasmids		
pGERM	pUC19-based suicide vector, Erm^R^	Shoemaker et al., 2000
pGREC	pGERM containing *recA* internal fragment	This study
pLYL01	Mob^+^, Tet^R^Amp^R^	Li et al., 1995
pLYLrecA	pLYL01 containing *B. fragilis recA*	This study

Rif, rifampicin; Gent, gentamicin; Erm, erythromycin; Tet, tetracycline; Strep, streptomycin; Amp, ampicillin; Mob, mobilisation; R, resistant; S, sensitive.

**Table 2:** Consensus sequences for regions of functional importance in the RecA protein.

Walker A	GPESSGKTT	Chen et al., 2007
Walker B	IVVD	Chen et al., 2007
L1/L2	EGDMGD FINQLREKIGVMFGNPETTTGGNALKFY	Chen et al., 2007
Glutamate (E)	IDAEHA	Bell, 2005
Glutamine (Q)	FINQL	Bell, 2005

**Table 3:** Primers used.

Name	Primer	Description	Reference
FRA	5′-GTA AAG CTG CAG ATG AAG TGA TCG-3′ (*Pst*I)	FRA and RRA amplify the full-length *B. fragilis recA* BF638R1245 gene. Restriction enzyme sites (in brackets) are underlined.	This study
RRA	5′-GGG CAT GCC TAT CGA GTT GG-3′ (*Sph*I)	This study
FBRT	5′-CCG GCT ATG ATC GGT GCC-3′	FBRT and RBRT amplify the intergenic region between BF638R1248 and BF638R1246/7.	This study
RBRT	5′-CGG CTT TAC GTA GCT CTG CG-3′	This study
RART	5′-CGT GGA TGG CCA GTG TCG-3′	FRA and RART amplify the intergenic region between BF638R1246/7 and BF638R1245.	This study
M13F	5′-CGC CAG GGT TTT CCC AGT CAC GAC-3′	M13F and M13R in combination with gene-specific primers allow verification of mutation in *B. fragilis* 638R *recA*.	Val Abratt Yanisch-Perron et al., 1985
M13R	5′-GAG CGG ATA ACA ATT TCA CAC AGG-3′	Val Abratt Yanisch-Perron et al., 1985
RIF	5′-CAG GTT CGA TAG CAC TGA ATG C-3′	RIF and RIR amplify an internal fragment of the *B. fragilis recA* BF638R1245 gene; used for the mutation of *recA*	This study
RIR	5′-CGG ATT ACC GAA CAT TAC ACC G-3′	This study

**Table 4:** Mtz susceptibility (MIC) of the *B. fragilis* strains.

Strain	MIC (mg/L)
638R	0.125
638R (pLYL01)	0.125
638R *recA*	0.016
638R *recA* (pLYL01)	0.016
638R *recA* (pLYL recA)	0.094
638R (pLYLrecA)	0.125
