# Corrigendum to: “*Bacteroides fragilis* RecA protein overexpression causes resistance to metronidazole.” [Res. Microbiol. 161 (5) (2010) 346–354]

**DOI:** 10.1016/j.resmic.2010.07.001

**Published:** 2015-06

**Authors:** Laura S. Steffens, Samantha Nicholson, Lynthia V. Paul, Carl Erik Nord, Sheila Patrick, Valerie R. Abratt

**Affiliations:** a Department of Molecular and Cell Biology, University of Cape Town, Private Bag, Rondebosch 7701, Cape Town, South Africa; b Karolinska Institute, Department of Laboratory Medicine, Division of Clinical Microbiology, Karolinska University Hospital, Stockholm, Sweden; c Centre for Infection and Immunity, School of Medicine, Dentistry and Biomedical Sciences, Queen’s University, Belfast, UK

The authors have pointed out that the symbols in the legend to Fig. 3, published earlier, were incorrect. The correct legend should read:

Fig. 3. Survival curves of the *B. fragilis* strains in response to DNA damage with (a) UV, (b) Metronidazole and (c) Hydrogen Peroxide. Filled diamonds, *B. fragilis* 638R(pLYL01); filled squares, *B. fragilis* 638R *recA^−^* mutant (pLYL01); filled triangles, *B. fragilis* 638R *recA^−^* mutant complemented with pLYLrecA; filled circles, *B. fragilis* 638R *recA* over-expressor (pLYLrecA). The errors bars represent the standard error calculated from at least three replicates of data.

The publisher regrets the error.

